# Evaluation of the scope, quality, and health literacy demand of Internet-based anal cancer information

**DOI:** 10.5195/jmla.2019.393

**Published:** 2019-10-01

**Authors:** Rebecca Charow, Michelle Snow, Sameera Fathima, Meredith E. Giuliani, Kate McEwan, Jordana Winegust, Janet Papadakos

**Affiliations:** Princess Margaret Cancer Centre, University Health Network, Toronto, ON, Canada, rebecca.charow@uhnresearch.ca; Princess Margaret Cancer Centre, University Health Network, Toronto, ON, Canada, michelle.snow@uhn.ca; Princess Margaret Cancer Centre, University Health Network, Toronto, ON, Canada, sameera09@gmail.com; Princess Margaret Cancer Centre, University Health Network, Toronto, ON, Canada, meredith.giuliani@rmp.uhn.on.ca; Princess Margaret Cancer Centre, University Health Network, Toronto, ON, Canada, highlandkate@sympatico.ca; Princess Margaret Cancer Centre, University Health Network, Toronto, ON, Canada, jordana.winegust@kidshelpphone.ca; Princess Margaret Cancer Centre, University Health Network, and Patient Education, Cancer Care Ontario, Toronto, ON, Canada, janet.papadakos@uhnresearch.ca

## Abstract

**Objectives:**

As there is a dearth of information about anal cancer available at cancer centers, patients often use the Internet to search for information. This is problematic, however, because the quality of information on the Internet is variable, and the health literacy demanded is higher than the average patrons’ capacity. The purposes of this study were to (1) determine the most common websites with anal cancer consumer health information, (2) identify the supportive care needs that each website addresses, and (3) evaluate the websites’ quality and health literacy demand.

**Methods:**

Medical Subject Headings (MeSH) entry terms for “Anus Neoplasms” were used in Google Canada to identify websites. Seven domains of supportive care needs were defined using Fitch’s Supportive Care Framework for Cancer Care. Website quality was evaluated using the DISCERN tool. Health literacy demand was assessed using readability calculators, where best practice dictates a grade 6 or lower, and the Patient Education Material Assessment Tool (PEMAT) that computes a percentage score in 2 domains, understandability and actionability, with 80% being an acceptable score.

**Results:**

Eighteen unique websites were evaluated. One website met health literacy best practices and had a “good” quality rating. Most websites addressed only 1 supportive care domain (61%), were of “fair” quality (67%), had readability scores higher than grade 6 (89%), and had PEMAT scores ranging from 41%–92% for understandability and 0–70% for actionability.

**Conclusion:**

The information gaps on anal cancer websites warrant a need for more health literate anal cancer health information on the Internet.

## INTRODUCTION

Anal cancer is a relatively uncommon cancer, but its global incidence is rising [1, 2]. The most recent statistics reported by the Canadian Cancer Society in 2013 state that approximately 580 Canadians are diagnosed with the disease each year, of whom 144 die as a result [3]. The American Cancer Society estimates that there will be 8,300 new cases of anal cancer in the United States in 2019, with 1,280 people dying from the disease [4]. However, due to advances in treatment, anal cancer is being transformed from a fatal disease to one that is increasingly curable [5]. Regardless of a curable or incurable cancer diagnosis, patients, families, and caregivers have significant information needs [6–8] that can be met with consumer health information and teaching from health care providers [9, 10].

Despite the efforts that consumer health librarians make to develop and maintain hospital library collections that are high quality for the diverse patient populations they serve, gaps exist in collections on rare diseases. In the instance of anal cancer, print patient information and educational resources are scarce at cancer centres in Canada. Specialty societies do not actively develop and disseminate anal cancer–specific print materials but do provide some information online [11]. As a result, when patrons seek information about anal cancer, consumer health librarians and library volunteers search the Internet to find information on an ad hoc basis.

Ad hoc searching is problematic for three primary reasons. First, although library volunteers are trained, they might not have the skills and experience needed to vet the quality of the information that they find online [12]. This is particularly concerning because the quality of online health information is highly variable, the information is often incomplete, and frequently the information is not what patients need to make well-informed decisions [13, 14]. Moreover, ad hoc searching for information can mean that patrons receive information that does not meet the quality standards of the library.

Second, ad hoc searching may unintentionally limit the scope of information that is available to patrons, because patrons who have the benefit of browsing a library collection can see what other information they might need [15]. In the case of cancer patients and their families or caregivers, these informational needs may be diverse, as supportive care needs extend far beyond pain or symptom management and counselling [16]. If patrons have to ask for information, they might not think to ask for information that falls in alternative domains. Therefore, ad hoc searching necessitates more deliberate search strategies, and patrons may not be aware of resource topics that could be beneficial to them.

Third, a significant proportion of Canadians have low health literacy [17], and individuals with low health literacy have difficulty accessing health information, appraising its quality, and applying it to their own circumstances [18]. As such, it is likely that a large proportion of patrons will not have the skills to assess the quality and applicability of health information themselves. Accessing reliable information on rare cancers, like anal cancer, is especially challenging since there is a lack of consumer health information due to the relatively small number of patients who are affected [19].

Furthermore, it is important to take a “universal precautions approach,” whereby services in health systems operate at a level where the health literacy demand is not strenuous for patients, families, and caregivers, because the assumption is all service users might have low health literacy [20]. A balance must be struck between the demands and complexities of the health system and the skills and abilities of its users [21]. To mitigate health literacy demand, plain language is one patient education strategy in which resources are written and formatted for the intended audience so that information is clearly understood and actionable [22, 23].

Considering the dearth of anal cancer resources in library collections, the global increase in anal cancer incidence [1, 2], advances in treatment [5], and the necessity for high-quality health-literate consumer health information [10], the authors sought to determine the breadth and quality of anal cancer consumer health information available on the Internet. The objectives of this study were to (1) identify the most common websites with information about anal cancer, (2) determine the supportive care needs addressed by anal cancer information found online, and (3) evaluate the quality and health literacy demand of anal cancer information found online. We further sought to establish a process to evaluate web-based consumer health information that could be reproduced by medical librarians who are interested in evaluating the quality of web-based consumer health information on other topics, including other rare diseases.

## METHODS

### Identifying websites with information about anal cancer

Entry terms for “Anus Neoplasms” in Medical Subject Headings (MeSH), the National Library of Medicine controlled vocabulary thesaurus for indexing articles, were used to determine the differing variations to communicate “anal cancer” in a systematic and repeatable manner. This resulted in ten MeSH-based search terms to identify websites for this evaluation: anal cancer; anal cancers; cancer, anal; cancers, anal; cancer of the anus; cancers of anus; anus cancer; anus cancers; anal neoplasm; and anus neoplasm. Although “anal neoplasm” and “anus neoplasm” are terms typically used by health care practitioners, they were included to account for patients, families, or caregivers who might use these search terms if they had heard or seen the terms in health-related reports.

Each search term was entered separately in Google Canada, and the first 15 websites that met the inclusion criteria were recorded in an Excel database. Website gathering was conducted via Google as it leads the search market in North America, with a more than 90% market share [24], and has been shown to have the highest search efficiency for health information when compared to other search engines [25]. The first 15 websites were recorded in an Excel spreadsheet to reflect search engine results page (SERP) Google data. Average traffic percentages in Google reveal that 93% of searchers only look at and select a website to view from the first SERP [26, 27], whereas traffic drops by 95% for the second SERP and continues to decrease for subsequent pages [27].

Inclusion criteria were websites that provided consumer health information on anal cancer that were published in English. Exclusion criteria included websites that were blogs, news or magazine articles, videos, books, dictionary definitions, and research or academic articles, as well as websites that were not available in English, required a membership to view the content, or had no relevance to anal cancer.

Each search term had its own sheet in the Excel spreadsheet documenting the following information: (1) title of web page, (2) name of publishing organization, (3) type of publishing organization, (4) country of publishing organization, (5) uniform resource locator (URL), (6) date of search, and (7) observations or comments. Types of publishing organizations were classified based on industry: nonprofit or charity, (private or public) company or subsidiary, professional (medical) society, government agency, or higher education. We also had a sheet for “cumulative” results, in which all websites were entered and tallied based on the number of search terms that retrieved a particular website.

### Determining supportive care needs addressed

Using the Supportive Care Framework for Cancer Care [16], which categorizes the needs of cancer patients into seven domains, we documented which domains were addressed by each anal cancer website in a table. The seven domains are: (1) emotional needs: coping with anger, despair, fear, and hopelessness; (2) practical needs: navigating finances, childcare, housekeeping, and legal systems; (3) spiritual needs: reflecting on the meaning of life and the concept of pain and suffering; (4) social needs: balancing family, relationships, school, and work; (5) psychological needs: dealing with issues of self-worth, body image, and coping strategies; (6) physical needs: dealing with pain, nausea, vomiting, and fatigue; and (7) informational needs: informing the patient’s, family’s, or caregiver’s decision making and providing information that assists in skill acquisition [16]. Three independent reviewers conducted this categorization, compared findings to ensure accuracy, and reported the categorization that they agreed upon. If disagreement occurred between the reviewers, a fourth reviewer facilitated consensus.

### Evaluating the quality and health literacy demand of anal cancer information

#### Quality assessment

To assess the quality of the consumer health information presented in each website, the validated and freely available online DISCERN tool [28] was used to encourage reproducibility of our approach among medical librarians. This tool is designed specifically to assess the quality of consumer health information content on the Internet about treatment choices, has been designed to be used by anyone (e.g., patients, families or caregivers, health professionals), and has been widely used in academic literature [29–39].

The DISCERN tool consists of 16 questions scored on a numeric rating scale ranging from No (1 point) to Yes (5 points). Questions are divided into 3 sections: (1) reliability and trustworthiness of the source of information, (2) comprehensiveness of the information regarding treatment choice, and (3) overall quality of the website. Higher scores indicate better quality: overall scores less than 27 indicate very poor quality, 27–38 indicate poor quality, 39–56 indicate fair quality, 57–62 indicate good quality, and 63 and above indicate excellent quality [28–39]. Two independent reviewers conducted this assessment, compared findings to ensure accuracy, and reported the average of their cumulative scores.

#### Health literacy demand assessment

Regardless of how informative and accurate a website might be, if it is not readable or well presented, it may not be accessible to the intended audience and, thus, helpful to readers. Since the DISCERN tool exclusively examines what information a website has provided, we also assessed readability, understandability, and actionability.

The readability of each website’s content was assessed using readability calculators, which are validated tools that compute grade levels of text based on vocabulary complexity [40]. Each readability calculator uses a mathematical formula to compute grade levels based on the number of words in a sentence, the number of words that have more than 2 syllables, and the number of sentences in a 100-word passage. We reported the average grade level from 7 calculators—Flesch Reading Ease, Gunning Fog, Flesch-Kincaid Grade Level, Coleman-Liau Index, SMOG Index, Automated Readability Index, and Linsear Write Formula—for each web page using free, publicly available online software [41] to encourage reproducibility of our approach among medical librarians. Readability calculators vary in their lenience and subjectivity, so taking average scores from multiple calculators is considered a strong practice. Content entry followed the US Department of Health and Human Services guidelines for reliability [42]. Plain language best practices for readability were met if websites scored at a grade level of 6 or below [43, 44].

The understandability and actionability of each website’s content were assessed using the validated Patient Education Materials Assessment Tool (PEMAT) [45]. Understandability is defined as key messages of text being understood by persons of any background or health literacy level, and actionability is defined as whether persons of any background or health literacy level understand and distinguish any actions that they can take based on the presented text [45]. Since only the written content was assessed and not any multimedia (e.g., videos), we used the PEMAT-Print version. This measure consists of 26 questions that evaluate text based on 4 categories to capture understandability: (1) content, (2) literacy demand, (3) graphics, and (4) layout and typography. To account for actionability, these measures consist of how the actions were described in the text and to whom they were addressed. The PEMAT allocates a percentage score for understandability and actionability separately, with higher percentages indicating greater understandability or actionability of the text. As with the Supportive Care Needs categorization, the same 3 independent reviewers used the PEMAT-Print to evaluate the understandability and actionability of each website, compared findings, and reported the average of their cumulative scores. Plain language best practices were met if websites had an understandability and actionability score of 80% or greater [46].

## RESULTS

### Numeric summary

The search methodology resulted in 18 websites for this evaluation: American Cancer Society [47], American Society of Colon and Rectal Surgeons [48], American Society of Clinical Oncology’s CancerNet [49], American Society for Radiation Oncology’s Radiation Therapy (RT) Answers [50], Bowel Cancer Australia [51], Canadian Cancer Society [52], Cancer Council Victoria [53], Cancer Research UK [54], Healthline [55], Macmillan Cancer Support [56], Mayo Clinic [57], Medicine Net [58], National Cancer Institute [59], National Health Service [60], Patient.info [61], University of California, San Francisco’s UCSF Health [62], University of Pennsylvania’s Oncolink [63], and WebMD [64]. Most websites identified were hosted by nonprofit or charity organizations (39%) [47, 51–54, 56, 57] and organizations based in the United States (61%) [47–50, 55, 57–59, 62–64] (Table 1).

**Table 1:** Summary of website details

Characteristic	n	(%)
Type of organization		
Nonprofit/charity [47, 51–54, 56, 57]	7	(39%)
Company/subsidiary [55, 58, 61, 64]	4	(22%)
Professional society [48–50]	3	(17%)
Government agency [59, 60]	2	(11%)
Higher education [62, 63]	2	(11%)
Supportive care domain/need		
Informational [47–64]	18	(100%)
Social [47, 49, 50, 52–54, 56, 57, 59]	9	(50%)
Emotional [47, 49, 50, 52–54, 56, 59]	8	(44%)
Physical [47, 49, 50, 52–54, 56, 59]	8	(44%)
Practical [47, 49, 52–54, 56, 57, 59]	8	(44%)
Psychological [47, 49, 52, 53, 56, 59]	6	(33%)
Spiritual [47, 52, 56, 59]	4	(22%)
Country		
United States (US) [47–50, 55, 57–59, 62–64]	11	(61%)
United Kingdom (UK) [54, 56, 60, 61]	4	(22%)
Australia [51, 53]	2	(11%)
Canada [52]	1	(6%)
Website scope		
Single domain [48, 51, 55, 58, 60–64]	11	(61%)
Multiple domains [47, 48, 50, 52–54, 56, 57, 59]	9	(50%)

### Supportive care needs addressed

The American Cancer Society website [47], Medicine Net [58], and WebMD [64] appeared most frequently, being retrieved by all ten search terms; however, of these websites, only the American Cancer Society also had subject matter that addressed all seven supportive care domains (Table 2). The website that appeared least frequently was the American Society for Radiation Oncology’s RT Answers [50], which only appeared when the search term “anus cancers” was used, and it only addressed the informational supportive care domain.

**Table 2:** Website frequency and supportive care needs domains addressed

Type of organization	Website	Frequency in search		Domains of need addressed						

		n	(%)	Informational	Social	Emotional	Physical	Practical	Psychological	Spiritual
Nonprofit/charity	American Cancer Society [47]	10	(100%)	✓	✓	✓	✓	✓	✓	✓
	Cancer Research UK [54]	9	(90%)	✓	✓	✓	✓	✓		
	Mayo Clinic [57]	9	(90%)	✓	✓			✓		
	Cancer Council Victoria [53]	8	(80%)	✓	✓	✓	✓	✓	✓	
	Macmillan Cancer Support [56]	8	(80%)	✓	✓	✓	✓	✓	✓	✓
	Bowel Cancer Australia [51]	3	(30%)	✓						
	Canadian Cancer Society [52]	3	(30%)	✓	✓	✓	✓	✓	✓	✓
Company/subsidiary	Medicine Net [58]	10	(100%)	✓						
	WebMD [64]	10	(100%)	✓						
	Healthline [55]	3	(30%)	✓						
	Patient.info [61]	2	(20%)	✓						
Professional society	American Society of Colon and Rectal Surgeons [48]	9	(90%)	✓						
	American Society of Clinical Oncology’s CancerNet [49]	9	(90%)	✓	✓	✓	✓	✓	✓	
	American Society of Radiation Oncology’s RT Answers [50]	1	(10%)	✓	✓	✓	✓			
Government agency	National Cancer Institute [59]	9	(90%)	✓	✓	✓	✓	✓	✓	✓
	National Health Service [60]	9	(90%)	✓						
Higher education	University of California, San Francisco’s UCSF Health [62]	9	(90%)	✓						
	University of Pennsylvania’s Oncolink [63]	7	(70%)	✓						

Most websites solely addressed the “informational” supportive care domain [47–64]. Whereas websites from all types of organizations addressed the informational supportive care domain, only nonprofit or charity, professional society, and government agency websites addressed the social, emotional, physical, practical, and psychological supportive care domains. Less than half of the websites addressed spiritual needs (22%) [47, 52, 56, 59], psychological needs (33%) [47, 49, 52, 53, 56, 59], practical needs (44%) [47, 49, 52–54, 56, 57, 59], physical needs (44%) [47, 49, 50, 52–54, 56, 59], or emotional needs (44%) [47, 49, 50, 52–54, 56, 59]. Only 4 websites (22%) encompassed content that addressed all supportive care domains: American Cancer Society [47], Macmillan Cancer Support [56], Canadian Cancer Society [52], and National Cancer Institute [59].

### Quality and health literacy demand

Overall, most websites were rated as being of “fair” quality (67%), with only the American Cancer Society website [47] receiving an “excellent” quality score of 66.5 (83%; SD: ±6.5; Table 3). Professional society websites had the highest average quality scores (50.8; 64%; SD: ±5.7) [48–50], followed by nonprofit or charity (46.9; 59%; SD: ±11.4) [47, 51–54, 56, 57]; government (45.8; 57%; SD: ±2.8) [59, 60]; higher education (41.0; 51%; SD: ±3.0) [62, 63]; and company or subsidiary (35.8; 45%; SD: ±8.6) [55, 58, 61, 64] websites. The website that received the lowest quality score was Healthline [55], which received a “very poor” quality rating (25.5; 32%; SD: ±2.5; Table 3).

**Table 3:** Evaluation of website quality and health literacy demand

Type of organization	Website	Quality		Readability (grade level)	Understandability		Actionability	
	
		DISCERN score	(SD)		PEMAT score	(SD)	PEMAT score	(SD)
Nonprofit/charity	American Cancer Society [47]	66.5	(6.5)*	8	70.3%	(21%)	38.7%	(8%)
	Cancer Research UK [54]	56	(3.0)	5†	87.7%	(13%)†	55.7%	(11%)
	Mayo Clinic [57]	41.5	(9.5)	8	76.3%	(15%)	70.3%	(8%)
	Cancer Council Victoria [53]	38.5	(0.5)	8	76.3%	(7%)	33.3%	(25%)
	Macmillan Cancer Support [56]	51	(10.0)	6†	73.0%	(7%)	60.0%	(0)
	Bowel Cancer Australia [51]	29	(6.0)	9	73.3%	(11%)	12.3%	(9%)
	Canadian Cancer Society [52]	46	(7.0)	7	74.3%	(20%)	46.7%	(15%)
Company/subsidiary	Medicine Net [58]	36.5	(12.0)	10	54.3%	(12%)	13.3%	(9%)
	WebMD [64]	32	(1.0)	10	56.7%	(4%)	6.7%	(9%)
	Healthline [55]	25.5	(2.5)	9	47.3%	(16%)	26.7%	(19%)
	Patient.info [61]	49	(3.0)	13	40.7%	(4%)	0.0	(0)
Professional society	American Society of Colon and Rectal Surgeons [48]	56.5	(4.5)	10	60.3%	(11%)	29.0%	(28%)
	American Society of Clinical Oncology’s CancerNet [49]	53	(5.0)	8	84.7%	(6%)†	53.3%	(19%)
	American Society of Radiation Oncology’s RT Answers [50]	43	(8.0)	11	55.0%	(11%)	36.7%	(12%)
Government agency	National Cancer Institute [59]	48.5	(4.5)	8	91.7%	(3%)†	41.0%	(30%)
	National Health Service [60]	43	(2.0)	10	76.0%	(13%)	26.7%	(19%)
Higher education	University of California, San Francisco’s UCSF Health [62]	38	(9.0)	11	49.7%	(14%)	26.7%	(25%)
	University of Pennsylvania’s Oncolink [63]	44	(3.0)	11	47.7%	(7%)	11.0%	(16%)

* Website of excellent quality.

† Meets plain language best practices: readability: grade level ≤6, understandability: ≥80%.

DISCERN items for which anal cancer websites fell short were: describing if no treatment is used (89% received a “very poor” score [49–64]); referencing areas of uncertainty (56% “very poor” [50, 52–57, 59, 62]; 22% “poor” [51, 52, 60, 63]); describing benefits of treatment (50% “very poor” [51, 54–59, 62, 64]; 39% “poor” [49, 50, 52, 53, 60, 61, 63]); describing how treatment choices affect quality of life (50% “very poor” [48, 51, 55, 58, 59, 61, 62–64]; 17% “poor” [50, 52, 60]); and providing support for shared decision making (44% “very poor” [50, 51, 55, 57, 58, 61, 62, 64]; 28% “poor” [48, 53, 54, 60, 63]) (Figure 1 in the supplemental appendix).

The health literacy demand of these websites was high, as only one website, Cancer Research UK [54], met plain language best practices. Cancer Research UK [54] had content written at a grade level of 5 and an understandability score of 87.7% (Table 3). Although 2 other websites also surpassed the best practices threshold of an 80% understandability score (American Society of Clinical Oncology [49] and National Cancer Institute [59]), their readability scores were high, at a grade level of 8. Readability scores for all websites ranged from a grade level of 5 to 13, with most websites having content written above a grade level of 6 (89%) [49–53, 55, 57–64].

The PEMAT understandability scores for all websites ranged from 41%–92%. The National Cancer Institute had the highest score (92%; SD: ±3%) [59], and Patient.info had the lowest score (41%; SD: ±4%) [61]. The PEMAT actionability scores for all websites ranged from 0–70%. The Mayo Clinic website had the highest actionability score (70%; SD: ±8%) [57], and Patient.info had the lowest score (0; SD: ±0) [61]. Overall, websites by companies or subsidiaries imposed the greatest health literacy demand on patients [55, 58, 61, 64]. On average, the readability scores of these websites were associated with a grade level of 11 (SD: ±1.5), the average understandability score was 49.8% (SD: ±6.3%), and the average actionability score was 11.7% (SD: ±9.9%).

## DISCUSSION

This evaluation demonstrates that most consumer health information websites about anal cancer are of low quality and require readers to have high health literacy. This is consistent with previous evaluations of online patient information for a diverse range of other medical conditions [31–38]. Only the Cancer Research UK website met plain language best practices and was rated “good” quality [54]. However, this website did not address all supportive care needs. Moreover, most websites did not address a breadth of supportive care needs, particularly spiritual or psychological needs. Most websites whose scope included all supportive care domains imposed a moderate health literacy demand and were of “fair” quality [52, 56, 59], except for one website that had an “excellent” quality score [47]. Websites by private and public companies or subsidiaries—such as Healthline, Patient.info, and WebMD—had the poorest quality, readability, understandability, and actionability [55, 58, 61, 64]. Therefore, our findings suggest that online anal cancer information is not accessible for most patients and families or caregivers, considering the prevalence of low health literacy.

When online information is accessible, patients report more informed decision making concerning health or treatment issues and greater willingness to make health behavior changes to improve health status [65]. The results of the present study indicate that understandable and actionable anal cancer resources are needed. This is likely the case for other rare cancers and conditions, which have fewer resources available due, in large part, to historically limited attention and investment [66].

Implications for medical librarians include: (1) collaborating with subject matter experts to develop understandable and actionable patient information about anal cancer; (2) advocating for evaluations of quality and health literacy demand before publishing any patient, family, and caregiver information online; and (3) utilizing this study to serve as a framework when developing Internet “pathfinder” documents for rare cancers, such as anal cancer, or other conditions for which there are gaps in library collections and internal resources are scarce [67]. In the case of anal cancer, these results can serve as guidance in place of ad hoc searching by providing a list of websites to recommend to patrons that are high quality, impose low health literacy demand, and address a range of supportive care needs.

A limitation of this study was that it utilized a convenience sample of websites (top 15 in the search yield) rather than sampling all websites. The drawbacks of convenience sampling are well documented and include lack of generalizability and insufficient power [68].

This study reveals several informational gaps in anal cancer websites and highlights a need for more understandable and actionable information. Most websites solely addressed the “informational” supportive care domain, with only four websites addressing all domains. Most websites had high health literacy demand, as they did not meet plain language best practices. The only website (Cancer Research UK) that met plain language best practices had a “good” quality rating and addressed five of the seven supportive care domains. This further reveals a need for comprehensive patient resources, particularly information pertaining to the spiritual and psychological needs of patients with anal cancer. Finally, this study provides a useful framework for identifying and evaluating the quality of consumer health information on the Internet and can be adapted to assess the quality and scope of informational websites for other cancer types.

## SUPPLEMENTAL FILE

Appendix **Figure 1** Average of each DISCERN item for website quality

## 

**Rebecca Charow**, rebecca.charow@uhnresearch.ca, Princess Margaret Cancer Centre, University Health Network, Toronto, ON, Canada

**Michelle Snow**, michelle.snow@uhn.ca, Princess Margaret Cancer Centre, University Health Network, Toronto, ON, Canada

**Sameera Fathima**, sameera09@gmail.com, Princess Margaret Cancer Centre, University Health Network, Toronto, ON, Canada

**Meredith E. Giuliani**, meredith.giuliani@rmp.uhn.on.ca, Princess Margaret Cancer Centre, University Health Network, Toronto, ON, Canada

**Kate McEwan**, highlandkate@sympatico.ca, Princess Margaret Cancer Centre, University Health Network, Toronto, ON, Canada

**Jordana Winegust**, jordana.winegust@kidshelpphone.ca, Princess Margaret Cancer Centre, University Health Network, Toronto, ON, Canada

**Janet Papadakos**, janet.papadakos@uhnresearch.ca, Princess Margaret Cancer Centre, University Health Network, and Patient Education, Cancer Care Ontario, Toronto, ON, Canada
