# Supplementary material for: Evaluation of the scope, quality, and health literacy demand of Internet-based anal cancer information
Source: J Med Libr Assoc. 2019 Oct 1;107(4):527–37. doi: 10.5195/jmla.2019.393 (PMC6774557; doi:10.5195/jmla.2019.393)

JMLA Journal of the Medical Library Association

Supplemental content to
J Med Libr Assoc. Oct;107(4):dx.doi.org/10.5195/jmla.2019.393
www.jmla.mlanet.org
© Charow, Snow, Fathima, Giuliani, McEwan, Winegust, Papadakos 2019

Evaluation of the scope, quality, and health literacy demand of Internet-based anal cancer information

Rebecca Charow; Michelle Snow; Sameera Fathima; Meredith E. Giuliani; Kate McEwan; Jordana Winegust; Janet Papadakos

APPENDIX

Figure 1 Average of each DISCERN item for website quality

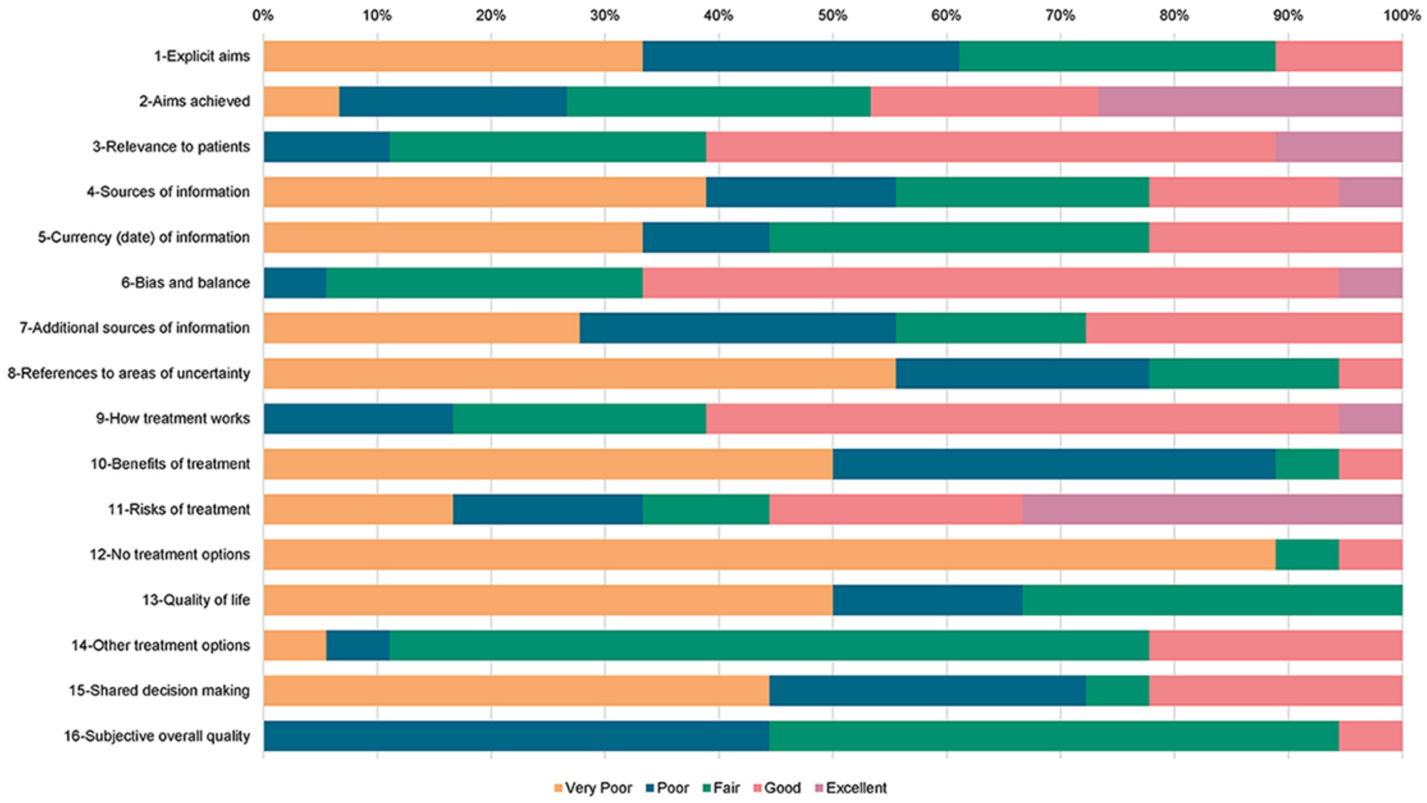

Supplement: Appendix [file jmla-107-527-s001.pdf]
